# Surface Temperature Simulation of Lunar Dayside and Its Geological Applications: A Case in Sinus Iridum

**DOI:** 10.3390/s19245545

**Published:** 2019-12-15

**Authors:** Jidong Zhang, Jinsong Ping, Zhaofa Zeng, Yongzhang Yang, Xiangyue Li, Mingyuan Wang

**Affiliations:** 1 College of Geoexploration Science and Technology, Jilin University, Changchun 130026, China; zhangjd14@mails.jlu.edu.cn (J.Z.); zengzf@jlu.edu.cn (Z.Z.); xiangyue17@mails.jlu.edu.cn (X.L.); 2 Key Laboratory of Lunar and Deep-Space Exploration, Chinese Academy of Sciences, Beijing 100101, China; wangmy@bao.ac.cn; 3 University of Chinese Academy of Sciences, No. 19(A) Yuquan Road, Shijingshan District, Beijing 100049, China; 4 State Key Laboratory of Information Engineering in Surveying, Mapping and Remote Sensing, Wuhan University, Wuhan 430070, China; yang.yongzhang@whu.edu.cn

**Keywords:** lunar surface temperature, Sinus Iridum, geological application, remote-sensing data

## Abstract

Lunar surface temperature is one of the fundamental thermophysical parameters of the lunar regolith, which is of great significance to the interpretation of remote-sensing thermal data. In this study, a daytime surface temperature model is established focusing on the lunar superficial layer with high spatial-temporal resolution. The physical parameters at the time of interest are adopted, including effective solar irradiance, lunar libration, large-scale topographic shading, and surrounding diffuse reflection. Thereafter, the 1/64° temperature distributions at five local times are quantitatively generated and analyzed in Sinus Iridum. Also, combined with Chang’E-2 microwave radiometer (CELMS) data and Diviner thermal infrared (TIR) data, the spectral emissivity distributions are estimated as a potential geological application of the simulated surface temperature. The results are as follows: (1) daytime surface temperature in Sinus Iridum is significantly affected by the local topography and observation time, and the influence of diffuse reflection energy is obvious; (2) the emissivity distributions provide a new way to understand the thermophysical properties difference of lunar regolith at different depths; (3) the influence of lunar orbiting revolution and precession on surface temperature should be analyzed carefully, which shows the importance of using the parameters at the time of interest.

## 1. Introduction

Lunar surface temperature is one of the important and fundamental parameters to interpret the thermal characteristics of the regolith from remote-sensing thermal data, which will give clues for solving thermal models and understanding the evolution of the Moon [1,2,3,4,5]. In particular, the lunar superficial layer temperature of the lunar regolith is not only regarded as a basic boundary condition for the thermal evolution models, but also represents the outermost thermal environment of the lunar surface, which changes a lot during a lunar day [6,7].

Thermophysical characteristics play important roles in understanding the lunar surface thermal environment and geological evolution [8,9,10,11,12]. Furthermore, remote-sensing thermal data is a key data source in geological interpretation. In order to eliminate the influence of observation time, the brightness temperature (TB) maps of the region of interest are usually generated at the same local time within a short time span [13,14]. The lunar orbital explorers, such as the microwave radiometer (CELMS) instrument onboard Chang’E (CE)-1/2 and Diviner instrument onboard the Lunar Reconnaissance Orbiter (LRO), have successfully measured the thermal emissions of the lunar surface. However, the orbital explorers’ measurements are limited by the instantaneous field of view of the detector at the observation time [15,16], thus, the remote-sensing data from different orbital explorers may not have the temperature information at corresponding observation time. Moreover, it may take a long time to obtain the same local time thermal distribution of the region of interest with high spatial-temporal resolution. Because the measurements from the orbital explorer are acquired in a very short time, the acquisition of lunar superficial layer temperature at the time of interest is not only indispensable in the in-situ lunar exploration but of great significance for the remote-sensing thermal data.

Great efforts have been made to obtain the lunar surface temperature for several decades [3,10,13,15,17,18,19,20]. The earliest method of lunar surface temperature detection was to observe the lunar surface by using ground-based infrared and radio telescopes [17,18]. Thereafter, orbital remotely-sensed observations and in-situ measurements enriched the ways and have obtained a lot of surface temperature information [3,10,13,15,19,20]. However, the ground-based observations can only measure the lunar near-side temperatures, and the spatial resolution is low. The cost of in situ measurements is too expensive to select many landing sites. In-orbiter measurements are limited by the satellite orbit and lack timeliness. Hence, how to overcome these problems becomes key in current lunar study.

Fortunately, with the help of thermal and physical properties of the lunar regolith acquired, the numerical simulation based on the theoretical model and regolith parameters emerges to meet the requirement. Because there is no atmospheric protection, the Moon is directly exposed to outer space. Compared with the night surface temperature, the daytime surface temperature is primarily influenced by the solar irradiance, surrounding thermal emissions, albedo and emissivity of the lunar regolith, which attracts more attention due to its complexity. Various lunar surface temperature models have been established in previous studies [2,3,4,5,7,21,22,23,24,25,26,27]. In the early time, the temperature models mainly employed the simplified variations of solar irradiance [21,22,23,24], which assumed that the temperature changed in a simple cosine function or Fourier series, and these simulations were not accurate enough because of the lack of topography and time-varying analysis. Thereafter, the scattering model within simple craters was preliminarily adopted based on the 1-D thermal model to acquire the temperatures in the lunar polar regions [25]. Based on the orbital parameters of the Moon and the Sun, the time-varying effective solar irradiance was gradually used to improve the lunar surface temperature model by means of analyzing the solar incidence angle and Sun-Moon distance [26,27]. In recent years, following with the acquisition of more detailed multi-source remote-sensing data, such as the Clementine Ultraviolet/Visible (UVVIS) data and Lunar Orbiter Laser Altimeter (LOLA) data, the solar albedo and large-scale topographic effect can be further introduced to simulate a more accurate surface temperature distribution [2,3,4,5,7]. Although these surface temperature models are constantly improved, these results are not combined with the physical parameters at the time of interest, and the effects of complex surrounding thermal environment and lunar libration are not mentioned.

Surface roughness is a main factor affecting the lunar thermal environment. It can cause a partial re-absorption of scattered or emitted radiation, and the surface temperature will increase with the self heating [28]. A significant limb brightening and unusually slow cooling of certain craters during lunar eclipse had been confirmed to be caused by surface roughness [29]. Moreover, most of the studies on the influence of surface roughness on surface temperature focused on the lunar polar regions to explore the water ice, which also showed the importance on the surface temperature [25,30]. In addition, because of the aspherical distribution of lunar mass, the rotational dynamics are not uniform [31]. Consequently, the influences of complex surface thermal environment and lunar libration on surface temperature need to be further considered.

In this paper, a surface energy balance is established to simulate the superficial layer temperature of the lunar regolith. Based on the 1/64° LOLA data and DE430 ephemeris, we improve the effective solar irradiance model and the diffuse reflection model, and the dayside surface temperature is simulated according to the physical parameters at the time of interest, which are different from previous works. Also, combined with the CELMS data and Diviner thermal infrared (TIR) data, the thermophysical feature difference in Sinus Iridum is investigated.

This paper is organized as follows. At the first step, a lunar surface temperature model is established and the physical parameters are discussed. Then, taking Sinus Iridum as the study area, five local-time temperature maps within 15 days are simulated and analyzed, and the uncertainty analysis is also included. After that, combined with CELMS data and Diviner TIR data, the spectral emissivity maps are estimated to reveal the thermophysical feature difference in Sinus Iridum, and the influence of observation time on the highest simulated temperature in 40 years is presented. Finally, the conclusions are given.

## 2. Methodology

The primary goal of this section is to predict the superficial layer temperature of the lunar dayside, therefore, we focus on the lunar surface boundary and solve this problem based on the surface energy balance.

### 2.1. A Physical Temperature Model

Taking an arbitrary lunar superficial layer as the boundary, there exists an instantaneous process of energy balance. From outside to inside, the energy includes direct solar radiation, Earth radiation, multiple scattering of solar radiation, surrounding infrared emissions, and the energy from the surface to the subsurface. From inside to outside, the energy includes the energy radiated from the lunar surface, and the heat flow inside the Moon. The thermal energy in both directions follows the energy conservation law. Based on the Stefan–Boltzmann rule, above relationship can be described by the following equation [7,32]:

(1) $$\left(1-\alpha )(I_{s}+I_{e}+I_{ref})+\varepsilon F+Q_{out}=\varepsilon \sigma T^{4}+K_{s}(\partial T/\partial x\right)$$

where α is the lunar surface albedo, *I_s_* is the direct effective solar irradiance, *I_e_* is the Earth irradiance, *I_ref_* is the multiple scattering of solar radiation, *ε* is the infrared surface emissivity, *F* is the thermal energy of surrounding infrared emission, *Q_out_* is the heat flow energy from the interior of the Moon to the surface. *σ* is the Stefan–Boltzmann constant, *T* is the superficial layer temperature, $K_{s}(\partial T/\partial x)$ is energy from the surface to the subsurface, *K_s_* is the surface conductivity.

Because the influences of *I_e_* and *Q_out_* on the surface temperature are both very small during the daytime [33], we neglect their contributions. Besides, it is reported that *K_s_* is very low [1,9,34], thus, the energy from the surface to the subsurface is almost 0 in a short time. Hence, we thoroughly analyze the other parameters to simulate the surface temperature with a better accuracy.

### 2.2. Parameter Identification

For the physical temperature model to simulate the lunar superficial layer temperature accurately, parameter identification must be discussed, including *I_s_*, *I_ref_*, *F*, and illumination conditions. These factors are all acquired at the time of interest.

#### 2.2.1. Improving *I_s_* Model

The *I_s_* model is the most important factor to predict the surface temperature of lunar dayside as a function of Sun-Moon distance (*D_sm_*) and incident angle of solar irradiance. When considering the perihelion and aphelion distances, the solar irradiance changes from 1326 to 1418 W/m^2^ [24]. The *I_s_* is expressed as follows [6]:

(2) $$I_{s}=I\cdot {\left(AU/D_{sm}\right)}^{2}\cos(z_{0})$$

where *I* is the solar constant and set to the average value of 1368 W/m^2^, *AU* is astronomical unit, *z*_0_ is the solar zenith angle in degree.

However, this model neglected the surface inclination, that is, *z*_0_ should be revised according to the relationship between position of the Sun and the surface topographic features. Fortunately, the position of the Moon and the Sun and lunar libration information at the time of interest can be inferred from the ephemeris. Hence, an improved geometric model about *z*_0_ is proposed based on topography data.

Slope and aspect are typically parameters to describe the relief and structure of the land surface based on raster data. In this paper, the vector-based surface geomorphic generation algorithm proposed in [35] is adopted, which is a widely used second-order, finite-difference algorithm using the elevation values of the four immediately adjacent pixels [36,37].

**Figure 1:**
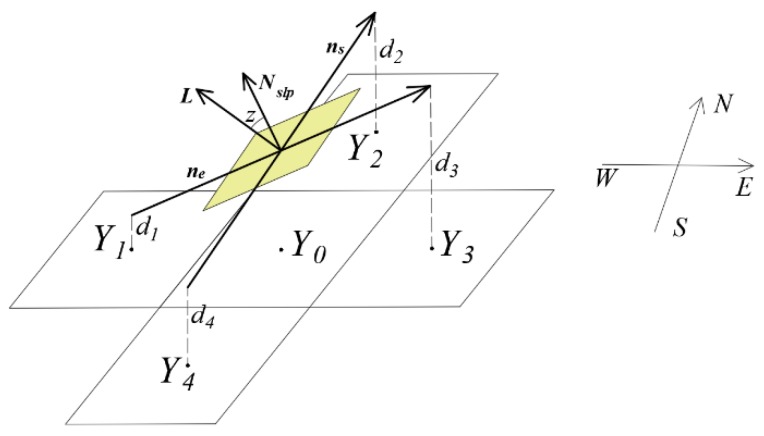
Schematic figure showing the relationship of the five pixel labeled *Y*_0_ through *Y*_4_ and geometric model of *z*.

As shown in Figure 1, five pixels are labeled *Y*_0_ through *Y*_4_. *Y*_0_ is the target site, and *Y*_1_ through *Y*_4_ are the four neighboring lunar unit surfaces of *Y*_0_, respectively. The dashed lines labeled *d*_1_ through *d*_4_ give the magnitude of the pixel elevations, rising from the pixel centers. The geocentric rectangular coordinate (*Yx_i_*, *Yy_i_*, *Yz_i_*, *i* = 1, 2, 3, 4) of the four pixels can be obtained from longitude, latitude, and elevation. Also, a vector with its tail at the center of pixel *Y*_1_, and its point at the center of *Y*_3_ is shown, which is denoted by ***n_e_*** (*Yx*_3_-*Yx*_1_, *Yy*_3_-*Yy*_1_, *Yz*_3_-*Yz*_1_), and gives an “east–west tilt” for *Y*_0_; similarly, the vector ***n_s_*** (*Yx*_2_-*Yx*_4_, *Yy*_2_-*Yy*_4_, *Yz*_2_-*Yz*_4_) from *Y*_4_ to *Y*_2_ gives the “north–south tilt” for *Y*_0_. The two vectors cross each other, forming a plane that is equivalent to the plane of *Y*_0_ (the yellow plane in Figure 1). The normal vector ***N_slp_*** produced by the cross product ***n_s_*** × ***n_e_*** is orthogonal to the plane of *Y*_0_, which can be used to describe the surface slope and aspect.

Based on the orbital parameters of the Moon and Sun at the time of interest, the vector ***L*** with its tail at the center of *Y*_0_ and its point at the center of the Sun is introduced. Furthermore, the included angle (*z*) between vector ***L*** and vector ***N_slp_*** can improve *z*_0_ respected to the horizontal plane and regard it as the solar incidence angle. The cosine value of *z* is the vertical component of ***L*** respecting to the plane of *Y*_0_, which can better eliminate the influence of surface slope on *I_s_*. It can be calculated by Equation (3):

(3) $$z=\arccos(N_{slp}\cdot L/(\left|N_{slp}\right|\cdot \left|L\right|))$$

Note that if *z* is greater than 90°, the direct solar illumination does not affect temperature, and cos(*z*) should be set to 0. Actually, *I_s_* is influenced by the Sun-*Y*_0_ distance (*R_sm_*), and it can be improved as follows:

(4) $$I_{s}=I{\left(AU/R_{sm}\right)}^{2}\cdot \cos(z)$$

#### 2.2.2. Acquisition of Illumination Condition

The illumination condition is another decisive factor to be further considered. The shadow areas can be determined by the solar illumination model [38,39], whether a target site can be illuminated depends on the value of *y* by using Equation (5):

(5) $$y=h_{sun}+\theta_{0}-h_{hormax}$$

where *y* is regarded as the Sun’s apparent angle, *h_sun_* is the elevation of the Sun; *θ*_0_ is the solar apparent radius; *h_hormax_* is the maximum value of the horizon elevation between the target site and front sites in solar azimuth direction. The target site is illuminated by the Sun only when *y* is greater than 0.

**Figure 2:**
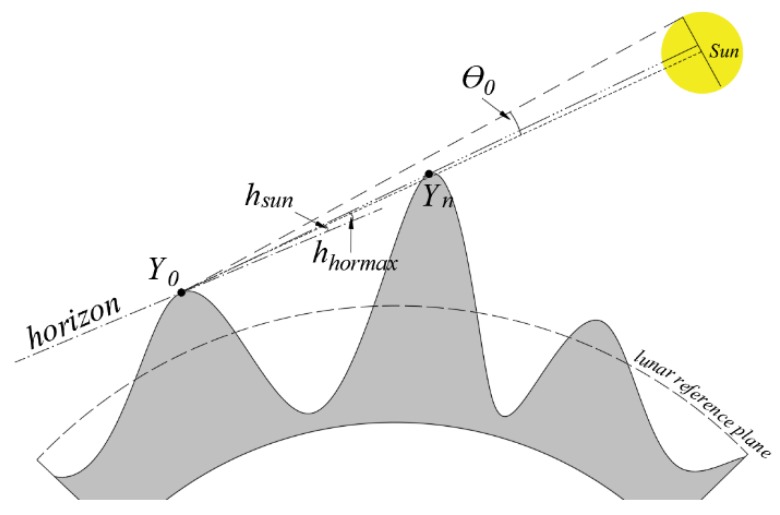
The target site *Y*_0_ illuminated by part of the Sun.

Furthermore, when the target site is illuminated by part of the Sun, the solar energy received will be greatly reduced. As shown in Figure 2, *Y*_0_ is the target site, *Y_n_* is the front site with the *h_hormax_*. When 2 × *θ*_0_ > *y* > 0, *Y*_0_ is only illuminated by part of the Sun. This introduced error on temperature should not be neglected especially near the lunar poles. Therefore, the visible fraction of the Sun (*P*) is introduced, which indicates the solar energy received as a percentage of the total energy emitted by the Sun. It can be expressed according to the area fraction of the solar disk:

(6) $$\begin{array}{cc} P=1 & y>2\times \theta_{0}\\ P=1-[\arccos(y/\theta_{0}-1)-\sqrt{2y/\theta_{0}-y^{2}/{\theta_{0}}^{2}}\times (y-\theta_{0})/\theta_{0}]/\pi & 2\times \theta_{0}\geq y\geq \theta_{0}\\ P=[\arccos(1-y/\theta_{0})-\sqrt{2y/\theta_{0}-y^{2}/{\theta_{0}}^{2}}\times (\theta_{0}-y)/\theta_{0}]/\pi & \theta_{0}>y\geq 0\\ P=0 & y<0 \end{array}$$

Specifically, *P* = 1, *Y*_0_ is illuminated by full of the Sun; *P* = 0, *Y*_0_ is in shadow; and in other cases, *Y*_0_ is illuminated by part of the Sun. Finally, *I_s_* is updated as follows:

(7) $$I_{s}=I{\left(AU/R_{sm}\right)}^{2}\cos(z)P$$

#### 2.2.3. Diffuse Reflection Energy

The diffuse reflection energy from surrounding lunar surface mainly includes the secondary reflection of direct solar radiation and thermal infrared radiation [32]. Evaluation of the diffuse reflection energy at the target site requires knowledge of the total flux scattered from or emitted by the surrounding lunar surfaces, as well as the fraction of this flux that actually reaches the target site. The fraction of energy that lunar unit surface emitted to the target site can be described mathematically by the view factor *β_j_* for a given surface topography [25], that is:

(8) $$\beta_{j}=\frac{1}{\pi }\cdot \frac{\cos\gamma_{0}\cos\gamma_{j}S_{j}}{{R_{j}}^{2}}$$

where *γ*_0_ and *γ_j_* are the angles between the surface normals of the target site *Y*_0_ and the lunar surface *j* and the line connecting their centers, *R_j_* is the distance between their centers, and the *S_j_* is the surface area of lunar surface *j*. Note that if *γ*_0_ ≥ 90° or *γ_j_* ≥ 90°, *β_j_* = 0. Moreover, there is a risk that surrounding surfaces is infinite, therefore, a limitation of *β_j_* is necessary. Here, the calculated radius is set to 5 km.

This model is easily calculated at simple landforms, such as the bowl-shaped craters, because all of the surrounding surfaces can transfer their energy to the target site. However, there is a crucial problem for complex terrain areas, that is, the line of sight between the target site and surrounding surfaces may be intercepted by topography. Therefore, the visibility must be analyzed. In the direction of line of sight, the target site will receive the surrounding energy only when the following relationship is satisfied:

(9) $${h_{j}}^{n}<h_{j}\;n\;=\;1,\;2,\;3,\;\ldots$$

where *h_j_* is the elevation between the target site to lunar surface *j*, *h_j_^n^* is the elevation of front sites between the target site to lunar surface *j*, *n* depends on the number of front sites.

(10) $$I_{ref}=\sum_{j=1}^{N}\beta_{j}\alpha_{j}I_{sj}k_{j}$$

(11) $$\left\{ \begin{array}{l} F=\varepsilon \sigma \sum_{j=1}^{N}\beta_{j}{{T^{\prime }}_{j}}^{4}k_{j}\\ {T^{\prime }}_{j}={\left[(1-\alpha_{j})I_{sj}/(\varepsilon \sigma )\right]}^{1/4} \end{array}\right.$$

where *N* is the total of surrounding lunar surfaces; *I_sj_* and *α_j_* are the effective solar irradiance and albedo of lunar surface *j*, respectively; *k_j_* = 1 represents visible, otherwise, *k_j_* = 0.

#### 2.2.4. Lunar Surface Albedo

Albedo α is related to the absorbed energy, and varies with the surface materials photometric properties. This parameter is considered as an empirical function as follows [40]:

(12) $$\alpha (z)=\alpha_{0}+a{\left(z/45\right)}^{3}+b{\left(z/90\right)}^{8}$$

where *α*_0_ is the normalized albedo, *z* is solar incidence angle, *a* and *b* are two empirical coefficients.

The Clementine UVVIS mosaic is a single 5-band image, which has been calibrated for the global Moon by radiometry, geometrical control, and photometry [41]. Furthermore, the 750-nm data are single-wavelength measurements, and a multiplicative factor of 1.31 is introduced to adjust the solar wavelength range [42]. This data has been considered in good agreement with the Diviner albedo and widely used in the surface temperature calculation [3,5,7]. In this paper, the albedo data derived from Clementine UVVIS 750 nm warped image mosaic is employed. The normalized albedo *α*_0_ can be converted from the image as below:

(13) $$\alpha_{0}=(DN\times 0.000137)/1.31$$

where *DN* = 16-bit pixel value of UVVIS image array. For the regions without data, the linear interpolation method is used to predict these values.

In addition, based on the global Diviner infrared data, the better estimated constant *a* = 0.06 and *b* = 0.25 in [4] are adopted here.

#### 2.2.5. Infrared Emissivity

The lunar surface temperature corresponds to black-body spectrum peaks in the middle to middle-far infrared range, thus, the emissivity varies with the wavelength. According to the sample measurements of Logan et al. [43] from Apollo 14 and 15 in the mid infrared and Perry et al. [44] from Apollo 11, 12, 14 and 15 in the far infrared, the emissivity is found to be generally higher than 0.9. Moreover, such studies are also estimated by Diviner TIR measurements and consistent with previous conclusions [1,45].

Among the studies of infrared emissivity, Bandfield et al. [45] acquired an apparent broadband hemispherical emissivity of 0.95 for the average daytime based on the Diviner emission phase function observations, which characterized the radiative behavior with wavelength and phase angle in detail. Moreover, this value is widely used in the study of surface temperature [3,4,5,32]. Therefore, ε = 0.95 is adopted in this study.

### 2.3. Temperature after Shading

For the complexity of topography, the lunar surface could be in shadow because of the terrain masking but not on regular sunset time. The surface temperature without the solar illumination can be regarded simply as a cooling process. Considering the accuracy and complexity, the relationship between the Apollo 15 heat-flow experiment (HFE) measurements and cooling time is empirically analyzed, and then its global application is discussed.

#### 2.3.1. Apollo 15 Heat-Flow Experiment (HFE) Data versus Cooling Time

The Apollo 15 HFE was conducted from 31 July 1971 to 31 December 1974. The experiment consisted of two probes to measure the temperature profile within the lunar regolith. Fortunately, the first thermocouple of probe 2, labeled TC1, is the nearest to the lunar surface, and the measured temperatures can be considered as the surface temperature [27]. Thus, the probe 2 night-time measurement is used to simulate the surface temperature variations in shadow.

By analyzing the illumination condition, Apollo 15 HFE data from the time just after sunset to 6:00 are selected. After preliminary data processing, 14,387 data are used to predict the surface temperature in cooling period, where the cooling time (*x*) is defined as:

(14) $$x=t_{e}-t_{s}$$

where *t_e_* is the observation time, *t_s_* is the time to start fitting, the unit of *x* is lunar hour.

**Figure 3:**
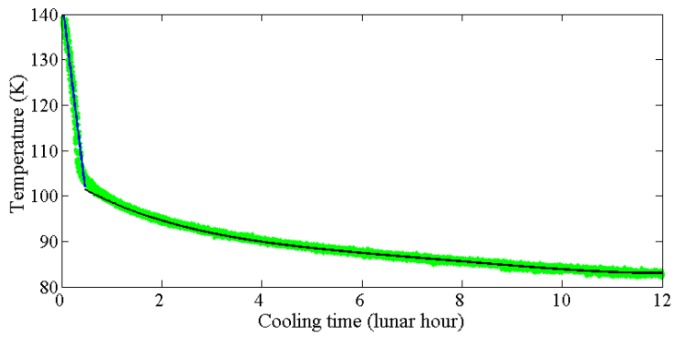
Apollo 15 heat-flow experiment (HFE) TC1 data (green dots) versus cooling time. The linear and the polynomial fitting models are in blue and black, respectively.

The Apollo 15 measurements are the green dots shown in Figure 3. According to the surface temperature distribution characteristics, the cooling period is roughly divided into two parts, the one is the first stage from 140 K in which temperature drops rapidly with time; the other is a slow descending stage with a small cooling rate. Thus, two fitting models are established.

From Figure 3, a linear fitting model is reasonable for the first stage, whereas how to divide the two parts is a key problem. We selected the Apollo 15 measurements greater than 105.5 K to calculate the cooling rate of the first stage due to the slope of the fitting model is the largest, −90.12 K per lunar hour.

In the slow descending stage, the temperature varies about 20 K, while the cooling time is close to 11.5 lunar hours. Therefore, a 4th-degree polynomial fitting model is acceptable to describe the temperature variation. In order to acquire a better connection between two models, when the temperature is greater than 102 K and less than 140 K, the linear model is adopted (blue line in Figure 3), where the fitting model is as below:

(15) T=140−90.12x

As shown in the black curve of Figure 3, when the temperature is lower than 102 K, the fitting model is as follows:

(16) $$T=0.003081{x_{1}}^{4}-0.09005{x_{1}}^{3}+0.9917{x_{1}}^{2}-5.79x_{1}+101.5$$

where *x*_1_ is the cooling time of temperature below 102 K, it can be expressed as Equation (17):

(17) $$x_{1}=x-38/90.12$$

#### 2.3.2. Global Application of Apollo 15 HFE Data

Due to the HFE only being conducted at one location, its global application should be further discussed. Previous works have shown that latitude dependence is an important typical feature of lunar surface temperature distribution [2,7,10]. To study the latitude variations of the simulated temperature, a fitting equation of the mean surface temperature was proposed and applied in [3]:

(18) $$T=T_{sur0}\cos^{0.32}(\phi )$$

where *T* is the surface temperature, *T_sur_*_0_ is the surface temperature at the lunar equator, ϕ is the selenographic latitude.

This model is based on 1D heat transfer equation and measured CE-2 CELMS data, and reveals latitude effect on the surface temperature. Therefore, this fitting equation is also adopted in this paper. Moreover, a relevant reasonable condition is that the simulated temperature variation in shadow can be adopted as the similar variation of Apollo 15 landing site entering the night. Thus, combined with the two fitting models, the approximate surface temperature in shadow at different latitudes can be acquired from the following equation:

(19) $$T_{lat}=T_{15}\times \cos{\left(\phi_{lat}\right)}^{0.32}/\cos{\left(\phi_{15}\right)}^{0.32}$$

where *T_lat_* is the surface temperature at the target latitude, *T*_15_ is the surface temperature from Equations (15) and (16), $\phi_{lat}$ is selenographic latitude of target site, $\phi_{15}$ is the selenographic latitude of Apollo 15 landing site. In addition, the initial fitting temperature also follows this rule.

## 3. Data Processing and Analyzing

Using the lunar surface temperature model, the same local time surface temperature maps are generated and analyzed in Sinus Iridum where is a typical region with abundant geomorphic features and complex evolutionary history. The LOLA dataset provides a variety of spatial resolution DEMs for the global Moon [46]. In this paper, the spatial resolution of the used LOLA data is 1/64°, which is high enough to simulate the surface topography and evaluate the geological applications of the simulated temperature.

### 3.1. Sinus Iridum

**Figure 4:**
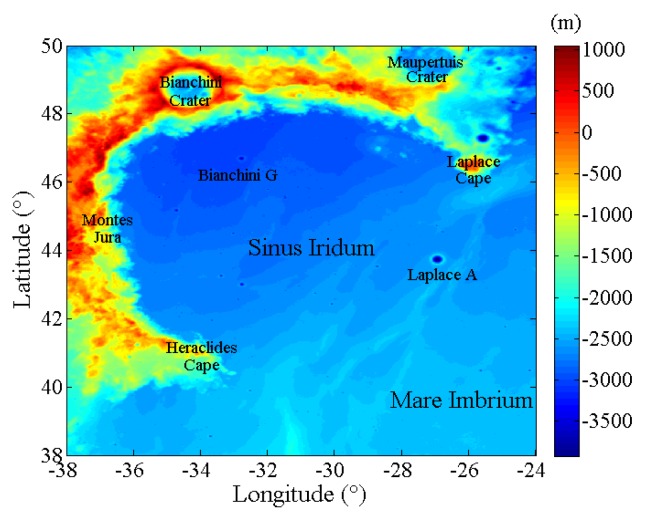
Elevation map of Sinus Iridum.

Sinus Iridum, centered at (45.01° N, 31.67° W), is about 249 km in diameter, which is an important bay in the northwest of the Mare Imbrium. As shown in Figure 4, the elevations in most area within Sinus Iridum are between −3000 and −2500 m and increase from the northwest to southeast in the floor. The geomorphologic features are abundant. It is surrounded from the northeast to the southwest by the Montes Jura range. Mare Imbrium is to the southeast of there through the Heraclides Cape and Laplace Cape. Also, there are extensive lava landforms in and around Sinus Iridum, there is reported to have a number of wrinkle ridges, but this topography is not obvious in the elevation map. In particular, the bay includes three important satellite craters: Maupertuis crater in the northeast, Bianchini crater in the north, and Laplace A crater along the eastern edge. There are also many small craters scattered at the inner Sinus Iridum, such as the Bianchini G crater.

Moreover, Sinus Iridum experienced several episodes of magmatic activity, indicating those of great geological significance. It has ever been recommended as the preliminary landing site of Chang’E-3 mission, and close to Luna 17 landing site. Based on the orbital image data, Schaber [47] distinguished three different basalts, which firstly showed the multiple magmatic activities within Sinus Iridum. Thereafter, Hiesinger et al. [48] divided the geological units and dated the basalt ages using Clementine UVVIS data, which again proved the complex and multi-stage evolutionary history. Chen et al. [49] interpreted the craters and stratigraphy of Sinus Iridum, showing the great differences in formation mechanism and age of the Mare Imbrium. Through analyzing the topographic, compositional, stratigraphic, and geological features of Sinus Iridum, Qiao et al. [50] defined the mare units and inferred their geological evolution history. Additionally, the geological and structural maps were studied by Wu et al. [51] with diverse remote-sensing datasets, the geology diversity further indicated the great importance of Sinus Iridum in understanding the lunar thermal evolution. Moreover, using CE-2 CELMS data, a microwave thermal perspective in Sinus Iridum was given by Meng et al. [11], which also validated the complex basaltic volcanism activities.

In summary, Sinus Iridum is of great significance in lunar geology research and worthy of further study. Therefore, the simulated temperature distribution maps are generated and hopefully enable researchers to understand these geological findings better.

### 3.2. Simulation Results

Based on the DE430 ephemeris data of the Jet Propulsion Laboratory (JPL) and the US Naval Observatory Vector Astrometry Software (NOVAS), the position of the Sun and the Moon and lunar libration can be obtained according to the time of interest. We compiled the corresponding calculation programs with the help of FORTRAN programming language.

**Figure 5:**
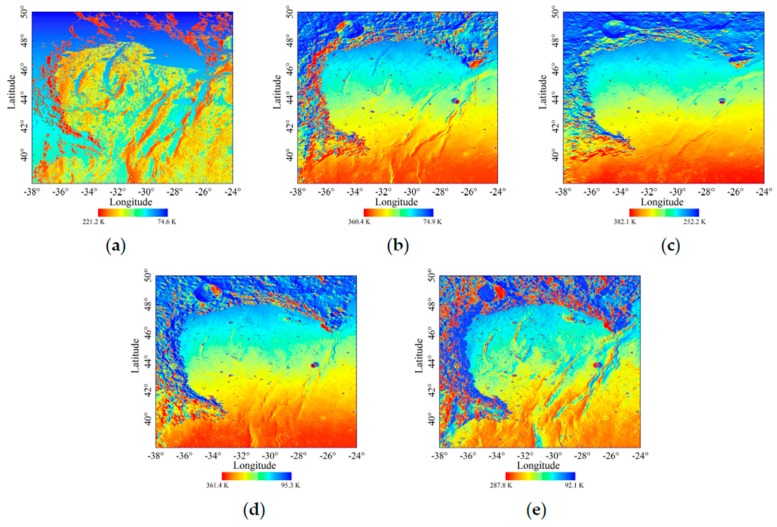
Surface temperature distribution maps of Sinus Iridum at 6:00 (**a**), 9:00 (**b**), 12:00 (**c**), 15:00 (**d**) and 17:30 (**e**).

As shown in Figure 5a–e, the temperature of every lunar unit surface in Sinus Iridum are generated at 6:00, 9:00, 12:00, 15:00 and 17:30, corresponding to the time span from 11 to 25 August 2019. In order to clearly show the mare temperature, the histogram equalization stretching method was used in the five maps. Generally, the thermal environment in Sinus Iridum is extreme, and the daytime temperature variation is displayed.

Figure 5a is the temperature distribution at sunrise when the elevation of the Sun is the lowest, which clearly reveals the great influence of topography. The result indicates the locations facing the Sun have obvious higher temperatures, especially at the the eastern part of the Montes Jura. The same characteristics appear at the west crater walls, such as the Bianchini crater and Maupertuis crater. The highest temperature of 221.2 K occurs at (35.96875° W, 41.875° N), where is the eastern boundary of the Montes Jura. There are large areas of the lowest temperature on the west side of the Montes Jura, the west side of the Laplace Cape, and the floors of Bianchini crater and Maupertuis crater, which enjoy longer nights. Interestingly, the wrinkle ridges greatly alter the surface temperature distributions when the elevation of the Sun is very low, and a more recognizable topographic feature is shown compared with the elevation map.

Similar features also occur at sunset. Figure 5e is the simulated temperature distribution at 17:30. The areas facing the west present a higher temperature, such as the west side of the Montes Jura and eastern inner wall of the Laplace A crater. Although the wrinkle ridges are not as obvious as at 06:00, the western mountain ridge of the Montes Jura is more clear, which again indicates the advantage of terrain recognition. Small craters within Sinus Iridum are clear, the temperature difference (Td) between the sunny crater wall and shady crater wall is greater. The highest simulated temperature occurs at the east inner wall of Laplace A crater, which is up to 287.8 K. Note that some regions have entered the night, such as the east side of the Montes Jura, where are in cooling time.

As shown in Figure 5b,d, when the elevation of the Sun is moderate, the simulated temperature increases significantly. There are similar simulated temperature distribution features in the morning and afternoon. Moreover, the simulated temperature distribution is less influenced by the topography, and the plains within Sinus Iridum and Mare Imbrium show the latitude effects. The low temperatures only scatter in several small craters and hillsides, and the mare regions present a higher temperature distribution.

Figure 5c is the simulated temperature distribution at noon, which is least influenced by the topography masking. Additionally, the temperatures of hillside, crater wall towards the equator and mountaintop are obviously higher than those of the arctic direction. The highest temperature is in the Laplace A crater, which is 382.1 K at the north crater wall, and the lowest temperature is 252.2 K at the south Bianchini crater wall. All the study areas are illuminated by the Sun, which shows that noon is less affected by the topography occlusion. The low simulated temperature areas appear on the shady slopes of some small deep craters. Furthermore, the temperature distribution within Sinus Iridum is obviously affected by latitude, which is about 15 K from 47° N to 40° N.

**Figure 6:**
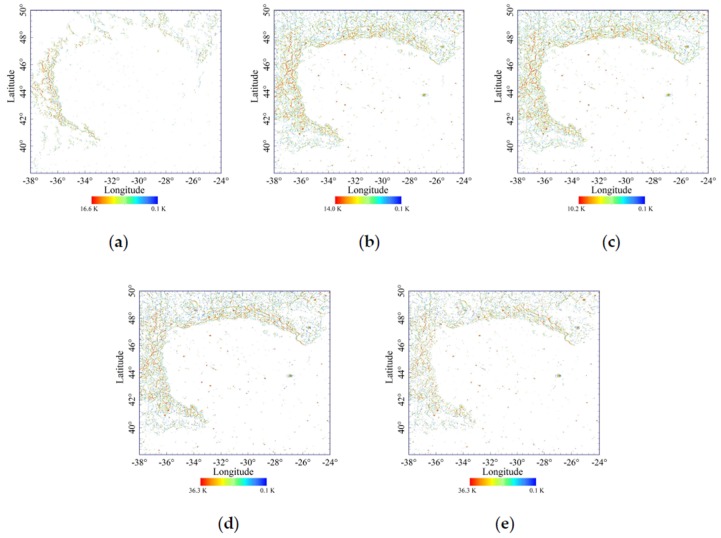
Temperature distribution caused by the diffuse reflection energy at 6:00 (**a**), 9:00 (**b**), 12:00 (**c**), 15:00 (**d**) and 17:30 (**e**), only the temperatures greater than 0.1 K are shown, and histogram equalization stretching is used.

Figure 6a–e are the temperature distributions caused by the diffuse reflection energy at five moments. In general, the effect of diffuse reflection is obvious, and the surrounding thermal energy will increase the temperature especially at the mountain valleys of the highlands and small crater rims. Diffuse reflection has little effect on most of the plain areas. The diffuse reflection temperatures at the mare regions are numerous and scattered. Although the direct solar irradiance is small when the elevation of the Sun is low, the highest temperature caused by scattering of solar radiation and surrounding infrared emission is higher. Moreover, although the highest diffuse reflection temperature is the lowest at noon, it is still about 10 K. The diffuse reflection mainly affects the temperatures of small craters at the mare regions, such as the Laplace A crater and Bianchini G crater. For the complex topography at the highlands, their influences vary greatly, especially when the elevation of the Sun is low. Hence, the temperatures caused by diffuse reflection energy should not be neglected.

### 3.3. Uncertainty Analysis

Above simulation maps present the characteristics of temperature variation in Sinus Iridum. In order to verify the uncertainty of the results, we compare with the Diviner data. The thermal emission energy acquired by Diviner can be inverted to describe the thermal emission features of the Moon. The telescope focal planes of Diviner are nine 21-element thermopile detector arrays [15]. Among its 9 channels, channel 7 (25–41 μm) is a broadband thermal infrared channel and of high signal-to-noise ratio. The spectral emissivity (ε_7_) of 0.98 is a representative value for everywhere [1]. Thus, the surface temperature can be converted from the infrared TB as follows [52]:

(20) $$T=T_{B}/{\varepsilon_{7}}^{0.25}$$

Diviner level 4 global cumulative products at channel 7 provide the thermal information at various local times. The Diviner data are the average calibrated TB binned at 0.5° latitude and longitude during about 6-year observations. According to its measuring time, the average temperature is calculated for comparison with a time step of 3 h from 5 July 2009 to 1 April 2015. In this paper, we select the location P1 (32.25° W, 44.25° N) in the mare regions and the location P2 (30.75° W, 49.25° N) at the highland for validation.

**Figure 7:**
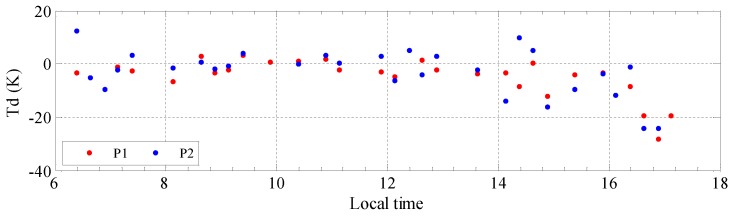
The Tds between the Diviner channel 7 data and the simulated temperature at P1 (32.25° W, 44.25° N) and P2 (30.75° W, 49.25° N). The Tds at P1 and P2 are in red and blue, respectively.

Figure 7 presents temperature differences (Tds) between the Diviner channel 7 data and the simulated temperature versus lunar local time. The results indicate a good agreement between the Diviner data and simulation results. Moreover, the Td variation near the noon is the most stable although the highest Td at P2 is about −6 K at 12:06. However, the Td in the afternoon is more obvious, more than 20 K. Considering the temperature model we used, with the decreasing of the elevation of the Sun, the solar irradiance may not the dominate factor affecting the surface temperature, and the heat from lunar subsurface layer should not be neglected. Therefore, our model can not handle with this condition very well, the results near the sunset are for reference only.

**Figure 8:**
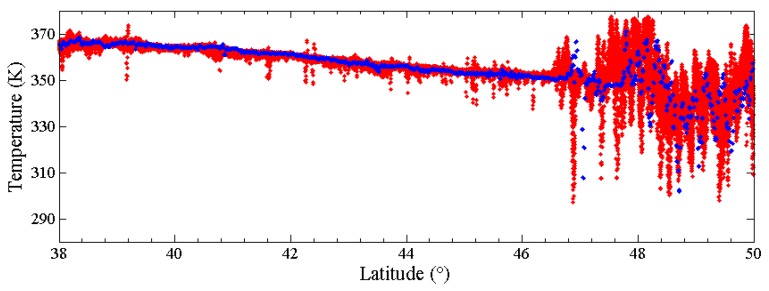
The simulated temperature versus the Diviner Reduced Data Record (RDR) channel 7 data at 00:40, 16 January 2017. The longitude is from 29.6° W to 28.4° W. The RDR data and simulated temperature are in red and blue, respectively.

The Reduced Data Record (RDR) data contain the record from the Diviner Lunar Radiometer Experiment collected during the orbital operations phase [53]. The RDR data include the observation time, channel number, detector number, the measured TB data, the local time, etc. In order to further verify the uncertainty temperature at noon, we select the RDR data measured on 00:40, 16 January 2017, and the local time is 12.58. The RDR data that we adopted records the TB from the 21 detectors of channel 7 at 1899 locations, and the longitude is from 29.6° W to 28.4° W. We simulate the temperatures at corresponding observation time. As shown in Figure 8, the surface temperatures are well simulated in the mare regions, and the latitude effect of surface temperature is clearly displayed. Although there are several inconsistencies at the highlands, most of temperatures are within the data range of the 21 detectors, and the variation trend is similar. Consequently, the result proves the rationality of our temperature model, and the simulated temperature at noon is good and suitable for studying its geological applications.

## 4. Discussions

Thermophysical features are strongly influenced by the temperature. However, the results revealed by orbital remotely-sensed observations are greatly limited by the deficiency of original data and the observation time. Therefore, the simulated temperature hopefully allows better understanding of the findings from these data related to the temperatures.

### 4.1. Geological Application with Remote-Sensing Data

Although the significance of surface temperature has been widely accepted, the observations in thermal infrared and microwave ranges are seldom to be fully considered using the temperature at the time of interest. Considering the parameters of lunar regolith are determined only by constants, inevitably these are influenced by different thermophysical features. However, these inconsistencies make it possible to distinguish different physical properties. Hence, the influence of latitude and topography should be eliminated.

The spectral emissivity (*e*) represents its effectiveness in emitting energy as thermal radiation, which is directly related to the material features [54]. The parameter is introduced and expressed as follows:

(21) $$e=T_{B}/T_{s}$$

where *T_B_* is the brightness temperature of the material, and *T_s_* is the physical temperature.

Here, we assume that the simulated temperature is approximately equal to the surface temperature. Therefore, the estimated emissivity derived by TB at different frequencies divided by simulated temperature can be use to understand the thermophysical difference of the lunar regolith [2,55]. Because the influence of latitude and topography will be greatly eliminated, and the composition and structure will play a more prominent role. Particularly, the simulated temperature at noon is used because of the best accuracy and less topographic influence.

The CELMS data and Diviner TIR data are two feasible data strongly related to temperatures, their geological applications are evaluated by the estimated spectral emissivity maps.

#### 4.1.1. Estimated Spectral Emissivity Features with Chang’E-2 Microwave Radiometer (CELMS) Data

The CELMS was the first instrument that measured the microwave thermal emission of the lunar surface at 3.0, 7.8, 19.35 and 37.0 GHz [16]. After the system calibration and geometric correction, all the data were processed at 2C-level, including the observation time, TB of four-channels, incidence angle and azimuth at sub-solar point, selenographic longitude and latitude, orbit altitude, and data quality state [8]. The CELMS data have the highest temperature sensitivity and spatial resolution, and can reveal different physical features of lunar regolith to a depth about 10 to 20 times of the used wavelength [56,57], whereas the application of the data is strongly limited. Therefore, we analyze the lunar regolith thermophysical properties by generating the emissivity maps to improve the application of the CELMS data collected from the CE-2 satellite.

**Figure 9:**
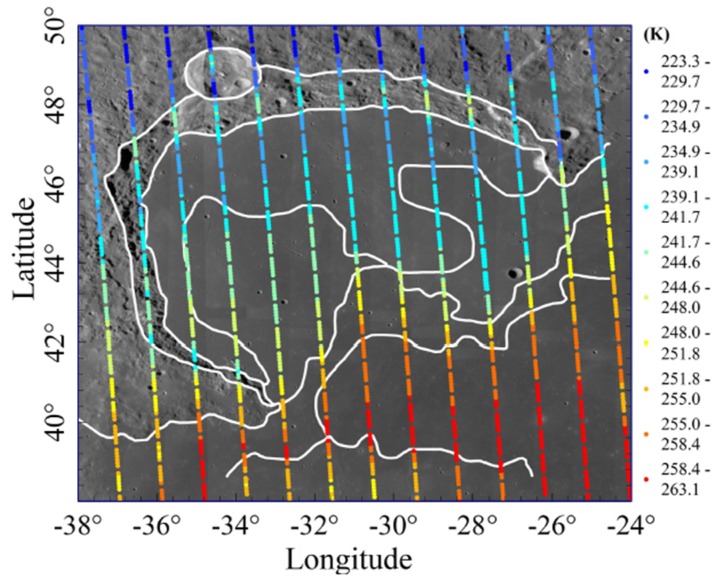
Scatter map of 37.0 GHz Chang’E-2 microwave radiometer (CELMS) data at noon overlaying on the Wide Angle Camera (WAC) image, The white line is the geological boundary interpreted in [50].

According to the range of the study area, 8424 CELMS data points at noon from the study area were selected after hour angle calculation [12]. Figure 9 is the scatter map of CELMS noon data at 37.0 GHz overlaying on the WAC (Wide Angle Camera) image, and each color point represents TB received by the CE-2 satellite at the observation point. It clearly indicates that the measurement is very high spatial resolution along the satellite orbit and about 1° spatial resolution between two neighbouring orbits. However, Figure 9 can not provide detail information about the lunar regolith thermophysical parameters. This is also one of the most crucial problems for the CELMS data applications.

The TB received by CELMS antenna is decided by the field of view. The spatial resolution of CE-2 CELMS data is 25 km at 3.0 GHz, and 15 km at other frequencies [3]. Hence, each cell can be modeled as a flat surface compared with the CELMS spatial resolution [58]. With these hypotheses, the CELMS data are processed as follows. Firstly, according the CELMS observation time, the average surface temperature in the field of view is calculated using the surface temperature model for every CELMS data points, which is simplified as the temperature within a spatial resolution. Secondly, the TBs of four channels are divided by the simulated temperature to acquire their estimated emissivities, respectively. Thirdly, the four-channel emissivities are interpolated by natural neighbor interpolating method to improve the spatial resolution, and the estimated spectral emissivity maps are generated with the spatial resolution of 0.25° × 0.25°.

**Figure 10:**
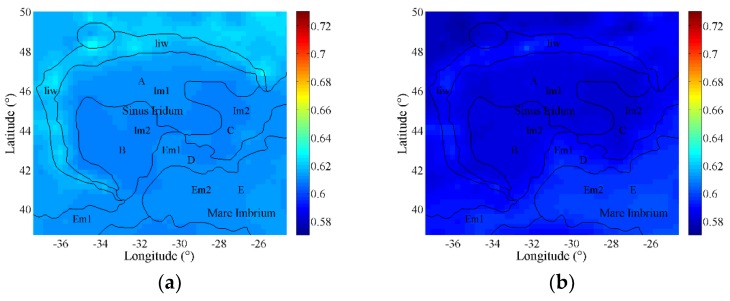

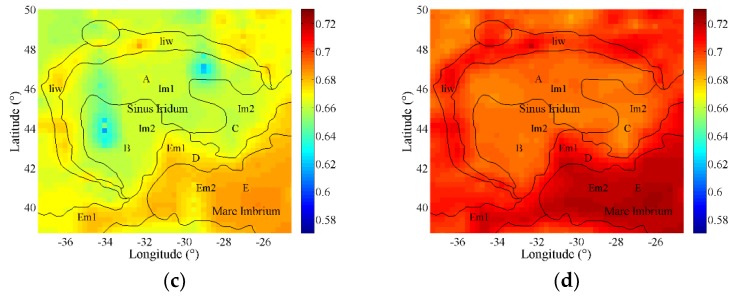
Estimated spectral emissivity maps in microwave range. (**a**) 3.0 GHz; (**b**) 7.8 GHz; (**c**) 19.35 GHz; (**d**) 37.0 GHz. The black line is the geological boundary in [50], and the five homogeneous units are represented by A–E. Im1 and Im2 are Imbrian mare stratigraphic units, and Em1 and Em2 are Eratosthenian mare stratigraphic units, liw is the wall materials.

The estimated emissivity maps are shown in Figure 10 using the same range. Interestingly, the estimated emissivity maps indicate the thermophysical property differences in emitting energy at four channels compared with the visible images. The geological differences are clearly presented among Sinus Iridum, Mare Imbrium, and the highlands.

The geological units of Sinus Iridum are analyzed in detail in [50], and in order to better understand the thermophysical features of the geological units, the interpretation results within Sinus Iridum and the northwest of Mare Imbrium are vectorized and overlaid on the emissivity maps in black line. As shown in Figure 10, there are five homogeneous units (A–E) in Sinus Iridum and northwestern Mare Imbrium region. The Im1 and Im2 are Imbrian mare stratigraphic units, and Im1 is older than Im2. The Em1 and Em2 are Eratosthenian mare stratigraphic units, and Em1 is older than Em2. The liw is the wall materials, which are hummocky to smooth material on moderate to steep slopes inside Iridum crater rim [47,49,50]. The outside of the wall materials at the highland distributed the rim materials, which was reported to be the debris ejected from the impact-formed Iridum crater and blanketed the older materials [47,49].

From Figure 10, the spectral emissivity distribution at highlands indicates the complex geological evolutionary history of Sinus Iridum. The highland can be divided into three parts according to the emissivity features. First, the wall materials were interpreted as the complex mixture of slumped Iridum ejecta and reworked by the Imbrium basin ejecta [47]. They always present a higher emissivity at four channels, indicating their higher effectiveness in emitting energy. Second, compared with the emissivity of wall materials, those of the north and northwest rim material are lower at four frequencies, and closer to those within Sinus Iridum. Third, the rim materials at the west and northeast of Sinus Iridum indicate higher emissivity and more complex distributions, which is more consistent with the geological interpretation results given in [49].

At the mare regions, the emissivity within Sinus Iridum is lower than those in the northwest of Mare Imbrium, indicating the different regolith thermophysical features. At the flat mare regions within Sinus Iridum, the emissivities are similar at low frequencies. The emissivities in unit A and unit B are almost consistent with each other at 3.0 GHz, but they are distinguishable at higher frequencies, which hints the change of the regolith thermophysical features with depth in Sinus Iridum. The emissivity in unit A is not so homogeneous. At the northwest within Sinus Iridum, the emissivity is low as well as the lowest elevation. There exists a higher emissivity at 37.0 GHz near the Bianchini G crater, indicating the inhomogeneous material composition might form in Copernian age. In addition, the emissivity near the foot of the Montes Jura in unit A is also higher than that in the northwest part, which shows the different material distributions when shaping Sinus Iridum. Unit B and unit C are both considered as the Eratosthenian unit, but the younger unit B presents a higher emissivity than that in unit C. Moreover, although the Laplace A crater locates in unit C, its emissivity is much higher than that in most parts of unit C and approximate to emissivity of the nearby wrinkle ridge in unit D. Questionably, there exists an obvious data anomaly within Sinus Iridum at 19.35 GHz, the emissivity distribution is quite different from those in other frequencies. According to the similar situation in previous works [8,11], the result at 19.35 GHz is only used for reference in this study.

Moreover, the emissivity performance of younger Eratosthenian-aged basalts (unit D and unit E) is apparently higher than the older Imbrian-aged basalts within Sinus Iridum. With the increase of frequency, the strata superposition at unit D and unit E is more obvious, and the younger stratum still presents a higher emissivity performance, which hints at the change of the regolith thermophysical features with depth.

**Figure 11:**
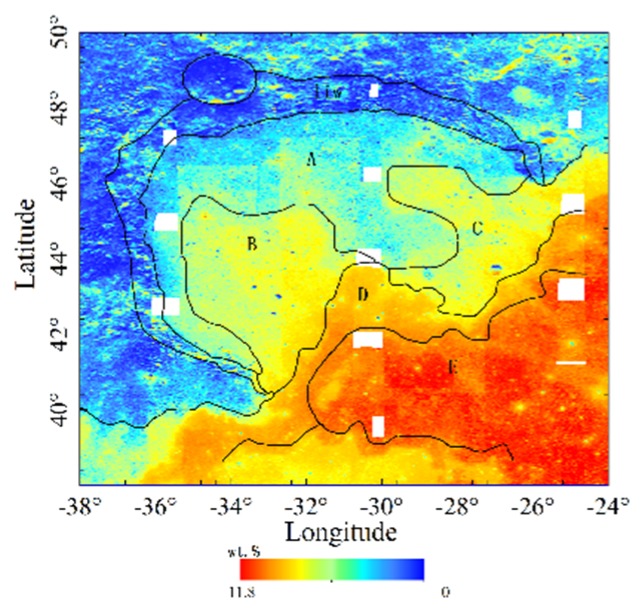
TiO_2_ abundance map of Sinus Iridum. The black line is the geological boundary in [50]. Histogram equalization stretching is used.

The relationship between the TiO_2_ abundance and the CE-2 CELMS data has been studied quantitatively in [5], which clearly indicates the great influence of TiO_2_ abundance on TB. As shown in Figure 11, TiO_2_ abundance map is inversed with Clementine UVVIS data, the oldest unit A has relatively low TiO_2_ contents, the younger unit B and unit C are slightly higher, and the youngest Eratosthenian-aged unit D and unit E have the highest TiO_2_ contents, which show that the emissivity features at high frequencies are mainly brought by the TiO_2_ abundance. The results also hint at the change of the regolith thermophysical features with depth in Sinus Iridum.

**Figure 12:**
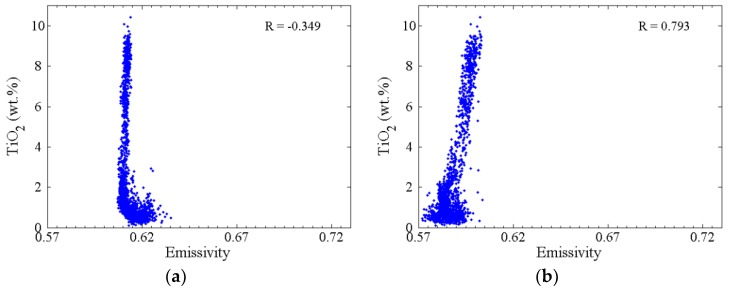

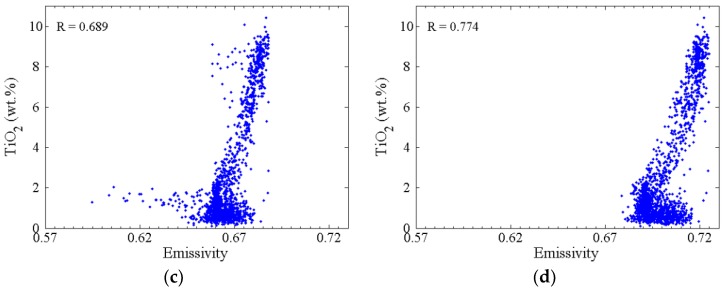
Estimated spectral emissivity versus TiO_2_ abundance of every CELMS data points, and R is the correlation coefficient. (**a**) 3.0 GHz; (**b**) 7.8 GHz; (**c**) 19.35 GHz; (**d**) 37.0 GHz.

Figure 12 is the scatter plot between TiO_2_ abundance and emissivity of every CELMS data points at four frequencies. In general, the correlation between low titanium and emissivities is not obvious. Except at 3.0 GHz, the high emissivity mostly corresponds to high TiO_2_ abundance. The correlation coefficient (R) is −0.349 at 3.0 GHz, but the Rs are 0.793, 0.689 and 0.774 at 7.8, 19.35 and 37.0 GHz, respectively. The results clearly show that there is a great influence of TiO_2_ abundance on microwave thermal emission at the shallower lunar regolith.

Compared with previous geological interpretation methods, the emissivity performance expresses the different geological units in Sinus Iridum well, and is of great significance for studying some potential geological issues.

#### 4.1.2. Estimated Spectral Emissivity Features with Diviner Thermal Infrared (TIR) Data

**Figure 13:**
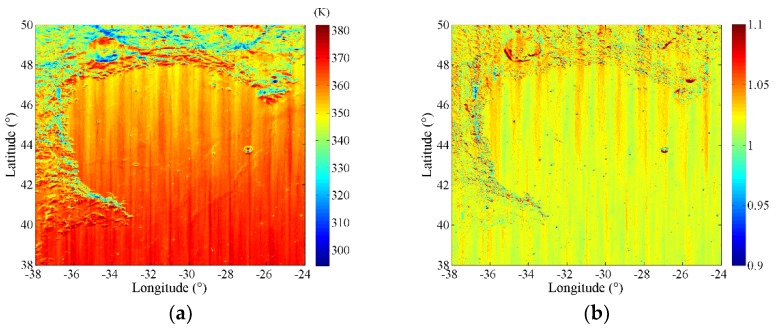
Infrared brightness temperature (TB) distribution of Sinus Iridum at 12:00 [14] (**a**) and estimated emissivity map using Diviner TB and the simulated temperature (**b**).

Diviner has systematically measured the solar reflections and infrared emissions of the whole Moon. For the deficiency of the original data, Diviner channel 6 (13–23 µm) was used to reveal the TB distributions of Sinus Iridum based on over 7-year measurements, the result at 12:00 is shown in Figure 13a, the spatial resolution is also 1/64° [14]. Although the differences of the highlands and the mare regions are shown properly, the recognition of flat mare regions is not as good. There are also some inconsistencies between the two results, such as the lowest simulated temperature and the temperature variations in complex terrain areas. This is not only due to the huge topographic fluctuation, but also brought by the great change of the temperature with the latitude and observation time. Furthermore, the stripe phenomenon caused by data anomaly in the push broom detection process of Diviner is noticeable. Nevertheless, this result provides us the infrared TB distribution reference with high spatial resolution, which is also a valuable reference for our study.

Figure 13b is the estimated emissivity map using Diviner TIR data and the simulated temperature. Here, we set the range of colorbar from 0.9 to 1.1 to increase contrast, which includes 99.8% of all data. Compared with Figure 13a, Figure 13b postulates that the change of the temperature with latitude is eliminated well, confirming the rationality of the simulated temperature. From Figure 13b, in general, the estimated emissivities at the mare regions are slightly lower than those at the highlands. Except the emissivities in the stripe regions, those at the south mare region is nearly the same as that at the north mare region. In addition, because the acquirement of the Diviner data is not in one time of a day but from the similar local time on different days, the emissivity difference resulted from the regolith thermophysical features is severely weakened due to different observation conditions in the stripe region. The results also imply that, the temporal and spatial resolution of Diviner data can be improved in the long-term observation, whereas the push-broom method inevitably leads to the lack of temporal and spatial coverage of the data over the whole Moon. Moreover, the emissivity behaviors in large craters as Bianchini and Maupertuis are similar as the nearby mountains, but not corresponding to the topographic features, implying the rationality of our model. However, there are some higher emissivities at the shady slopes than those in other regions, which indicates that the thermal environments is more complex than what we simulated.

In summary, compared with the Diviner data, the CELMS data can be used to evaluate the difference in thermophysical features of the deeper lunar regolith. The superficial layer of the lunar regolith may be greatly affected by other factors, such as strong space weathering. The estimated emissivity maps provide a reference of the effectiveness in emitting thermal energy of the lunar regolith at different depths.

### 4.2. Useful Supplementary Information

With the start of the CE-4 mission, the selection of the landing site has become increasingly popular in current lunar study. To select the landing site scientifically, the temperature distribution is always thought as the first important factor. Moreover, the Diviner TIR data proves the uncertainty due to the long term observation, however, this effect has not been studied quantitatively. Against this background, the study on temperature changed with time is a useful supplement in such work. Therefore, the temperature variations are quantitatively analyzed based on the temperature model.

It can be deduced from the foregoing that the motion of the Sun and the Moon will directly affect the parameters of *I_S_* in the temperature model. Hence, the lunar orbital motion should be seriously reconsidered. Although the previous studies showed the orbital period effect on temperature should not be ignored by reanalyzing the Apollo 15 and 17 measurements, the changes of the temperature based on lunar days have not been thoroughly studied [26,59]. The precession of 18.6 years and rotation of 29.5 Earth days are two typical periodic motions of the Moon, and we discuss the variation of the highest temperature in a lunar day from 2005 to 2045, which reveals the greatest influence of lunar motions on temperature.

**Figure 14:**
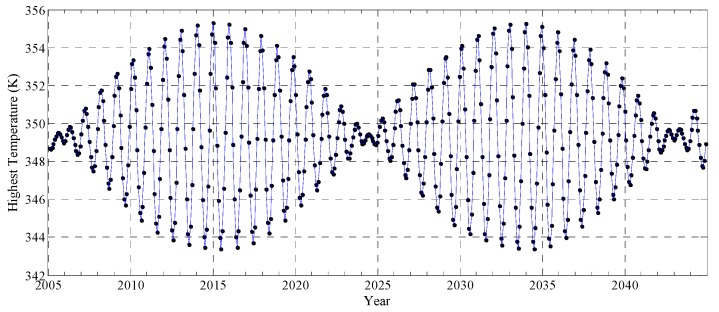
The variations of the highest temperature (black dots) in a lunar day at (32° W, 44° N) from 2005 to 2045.

Figure 14 is the variation of the highest temperature of a lunar day at (32° W, 44° N) for 40 years, which includes 494 lunar days. Figure 14 postulates that the highest temperature varies periodically with time, which can be concluded as having three characteristics.

Firstly, the change of the highest temperature ranges from 0 to 3.1 K in two neighboring lunar days. For example, in the 5th and 6th lunar day of 2024, the highest temperatures are both 349.4 K; however, the change of the highest temperature between the 3rd and 4th lunar day in 2034 is up to 3.1 K. These results indicate that the change of temperature can not be ignored even if two observations are at the nearest same local time, which is essentially significant for the application of the Diviner data.

Secondly, although there are also four seasons on the Moon in a year, the change of the temperature with season is apparent in different years. Specifically speaking, the greatest change of the highest temperature is up to 11.9 K in 2015, while this value is only about 1.2 K in 2042. This indicates that the influence of seasonal variations on temperature needs to be carefully analyzed in different years.

Thirdly, there occur two similar trends within the 40 years. Each period is about 19 years: the one is from 2005 to 2024, and the other is from 2024 to 2043. Particularly, the highest temperature in 2024 is 349.4 K, but that in 2015 is 355.3 K, the range of temperature is up to 5.9 K at half of the lunar precession. Also, the lowest temperature in 2024 is 348.9 K, but that is 343.4 K in 2015 and 2034, the Td is 4.5 K. These clearly show the influence of lunar precession on the temperature.

The results again prove that the temperature data collected by the probes in orbit are heavily affected by the observation time, even if in the same position and local time. This phenomenon has been proved by the Diviner TIR data used in Figure 13. Although there are small changes of the temperature in some years, the Td resulted from observation time should be analyzed carefully, especially using the long-term orbit temperature data. This also shows the advantage of the numerical simulation model.

## 5. Conclusions

In this paper, a daytime surface temperature model is established focusing on the lunar superficial layer with high spatial-temporal resolution. The lunar superficial layer temperature distributions of Sinus Iridum are simulated at five local times with a spatial resolution of 1/64°. Thereafter, combined with CELMS data and Diviner TIR data, the spectral emissivity distributions are estimated as a geological application of surface temperature. Finally, the importance of using the parameters at the time of interest is proved. The main results are as follows.

Firstly, the temperature model established in this study is based on the parameters at the time of interest, including the effective solar irradiance, large-scale topographic shading and slope, lunar libration and diffuse reflection energy from the thermal environment. The results simulated in Sinus Iridum show the effects of diffuse reflection, latitude and topography.

Secondly, the same local time temperature distribution plays an essential role in evaluating the regolith thermophysical parameters. The thermophysical properties difference in emitting energy may be expressed better using the estimated spectral emissivity maps combined with the CELMS data and Diviner TIR data.

Thirdly, the influence of observation time should be analyzed carefully when using the orbital remotely-sensed data related to temperatures. Lunar orbiting revolution and precession are both important factors affecting the surface temperature, and their effects vary with time.

The temperature model can be used to study the lunar regolith properties with other source data, and also provide an important reference for simulating the surface temperature of airless heavenly bodies. The temperature distribution with high spatiotemporal resolution should be further studied to reveal more geological issues.
